# Advances in cytokine gene polymorphisms in tuberculosis

**DOI:** 10.1128/msphere.00944-24

**Published:** 2025-03-31

**Authors:** Haiyang Fu, Wenqiang Sun, Ye Xu, Haiyun Zhang

**Affiliations:** 1 Affiliated Nantong Hospital of Shanghai University (The Sixth People's Hospital of Nantong), Jiangsu, China; 2 Department of Kidney Transplantation, Center of Organ Transplantation, The Second Xiangya Hospital of Central South University, Changsha, Hunan, China; 3 Department of Laboratory, Dalian Municipal Women and Children’s Medical Center, Dalian, Liaoning, China; Washington University in St. Louis School of Medicine, St. Louis, Missouri, USA

**Keywords:** pulmonary tuberculosis, susceptibility, genetic polymorphism, single nucleotide polymorphism

## Abstract

Tuberculosis (TB), especially pulmonary tuberculosis (PTB), is a prevalent infectious disease affecting the respiratory system and is characterized by high morbidity, disability, and mortality rates that significantly impact the quality of life of patients and their families. Host genetic susceptibility plays a crucial role in the infection process of *Mycobacterium tuberculosis* (*M. tuberculosis*) with single nucleotide polymorphisms (SNPs) identified as key factors in the genetic loci associated with tuberculosis occurrence and progression. Research indicates that polymorphisms in cytokine genes—including interferons, interleukins, tumor necrosis factors, and chemokines—are closely linked to the onset, progression, and treatment outcomes of pulmonary tuberculosis. Investigating cytokine gene polymorphisms in PTB patients is essential for understanding disease mechanisms and prognosis. This review summarizes the role of cytokine polymorphisms in tuberculosis morbidity, elucidates the biological genetic mechanisms involved at the molecular level, and provides insights into clinical treatment strategies for TB.

## INTRODUCTION

Single nucleotide polymorphisms (SNPs) are among the most common heritable variations in humans and are defined as deoxyribonucleic acid (DNA) sequence polymorphisms arising from single nucleotide variations due to transitions, inversions, insertions, or deletions of a single base, making them the most abundant form of genetic variation (1). SNPs are prevalent in the human genome, constituting more than 90% of all variations in human genomic DNA, with an average of one SNP per thousand bases (2, 3). With respect to genomic variation, SNPs have two functions: first, as genetic molecular markers (4); second, to elucidate the relationship between genes and diseases, facilitating the identification of relevant genes during disease onset (5, 6). SNPs located in coding regions can influence the structure and function of proteins, whereas SNPs in noncoding regions are more frequently observed as molecular markers of disease (7). Exploring the characteristics of complex polygenic genetic diseases through SNP information represents a significant challenge in biology.

Tuberculosis (TB) is a chronic infectious disease caused by *Mycobacterium tuberculosis* (*M. tuberculosis*), a member of the *Mycobacterium tuberculosis complex*. TB remains a significant global public health challenge due to emerging drug resistance and prolonged treatment durations (8). The Global Tuberculosis Report 2022 issued by the World Health Organization (WHO) points out that there will be about 10.6 million new TB patients in the world in 2021, the incidence rate of TB will reach 134/100,000, and the death toll of TB will reach 1.6 million in 2021 (9). Moreover, The global burden of drug-resistant TB remains high. According to the WHO’s Global Tuberculosis Report 2023: 175650 multidrug-resistant TB (MDR-TB) patients were included in treatment globally, accounting for about one-third of the patients who need treatment, and the treatment success rate was 63% (10). In 2019, the median per capita treatment cost of sensitive tuberculosis patients was 860 US dollars, while that of MDR-TB was 5,659 US dollars. The treatment cost of MDR-TB was more than six times that of drug-sensitive TB (11). The current situation regarding TB prevention and control is quite severe (12). Research on tuberculosis prevention and control has once again emerged as a prominent focus both domestically and internationally.

**Fig 1:**
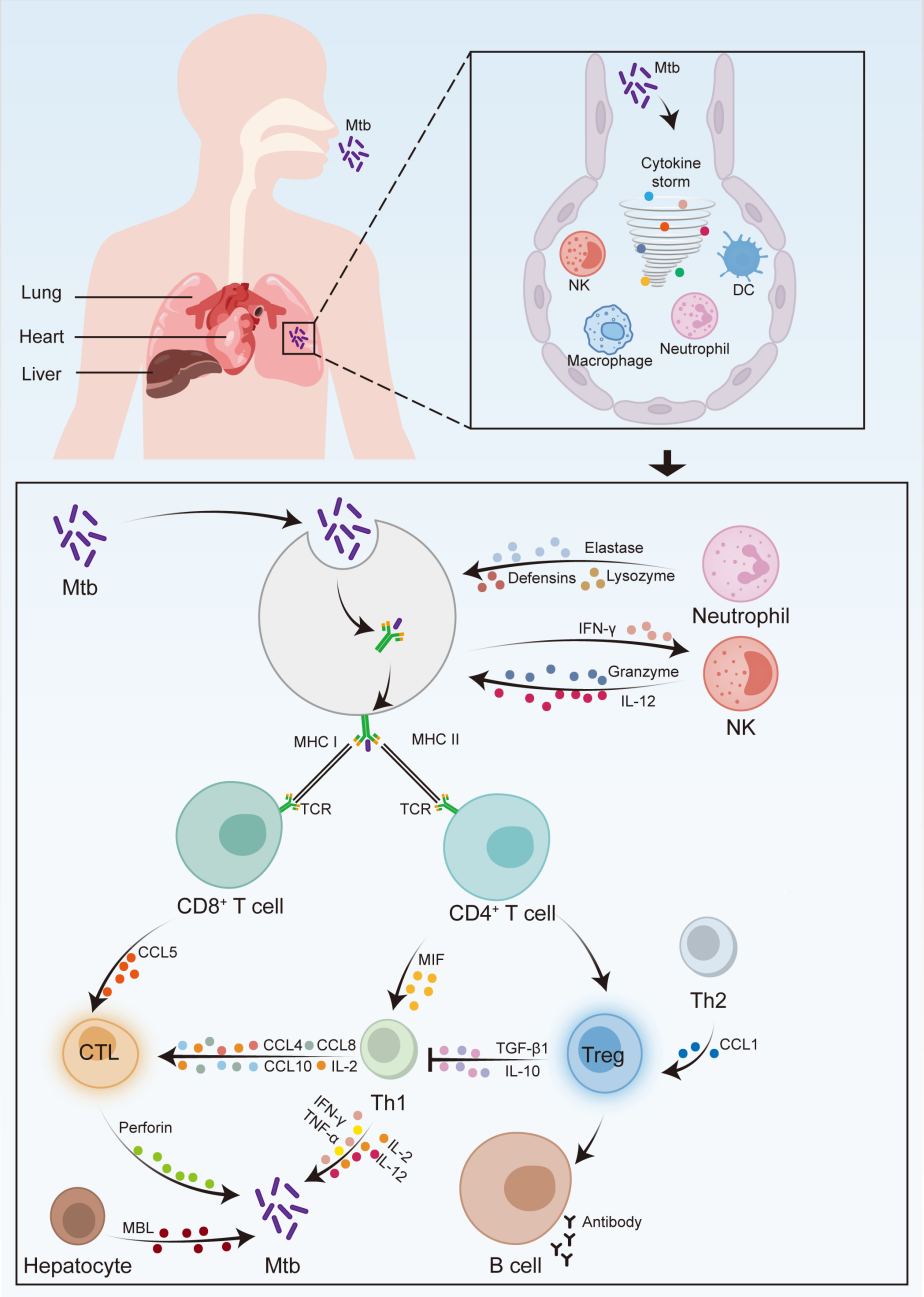
Schematic diagram of the immune response induced by Mycobacterium tuberculosis (Mtb) infection in humans. (**A**) Anatomical overview of the human body. It is demonstrated that the primary transmission route of Mtb is via the respiratory tract, and the lung is the principal organ involved in Mtb infection. (**B**) Detailed illustration of the lung and immune system. An enlarged depiction of the lung microenvironment shows the interactions between Mtb and various immune cells. Upon entering the alveoli through the respiratory tract, Mtb is phagocytosed by macrophages, resulting in the release of cytokines. Dendritic cells (DCs) capture Mtb antigens and migrate to lymph nodes to activate T cells. Natural killer (NK) cells and neutrophils are depicted as interacting with infected macrophages. (**C**) Interactions among immune system cells and molecules. A detailed view of the immune response cascade begins with the activation of CD8^+^ T cells and CD4^+^ T cells. CD8^+^ T cells differentiate into cytotoxic T lymphocytes (CTLs), which release perforin and granzyme to eliminate infected cells. CD4^+^ T cells differentiate into Th1, Th2, and Treg cells. Th1 cells produce interferon-γ (IFN-γ) to activate macrophages. Th2 cells produce cytokines that promote B cell activation and antibody production. Treg cells produce IL-10 and transforming growth factor-β1 (TGF-β1) to regulate the immune response. Arrows and labels indicate the direction of cell movement and the release of cytokines and other immune mediators. This schematic diagram uses color coding to distinguish between different cell types and immune mediators.

*M. tuberculosis* invades the human body, primarily by residing within alveolar macrophages (Fig. 1). Although unactivated phagocytes can phagocytose *M. tuberculosis*, they often struggle to eliminate it. The body’s response to *M. tuberculosis* infection is predominantly mediated by CD4^+^ T-cell-mediated cellular immunity, while intrinsic immunity and cytokines secreted by the body also play significant roles (13, 14). Cytokines are released primarily by lymphocytes and monocyte-derived macrophages and are integral to inflammatory and immune responses. Cytokine synthesis and levels are subject to genetic regulation (15, 16). Genetic variation in immune-related genes has been shown to influence host resistance to *M. tuberculosis* infection, disease severity, and drug resistance (17). Variations in susceptibility to TB may arise from the expression of genetic polymorphisms in humans (18). Given the increasing number of TB patients, increasing antimicrobial resistance, and prolonged treatment durations, novel treatment modalities must be developed. Currently, association studies of candidate gene polymorphisms are frequently employed to investigate genetic susceptibility to TB, with SNPs representing the most common form of genetic polymorphism. SNPs are widely utilized as clinical markers due to their high density, conservation, genetic stability, and ease of typing. They are crucial determinants of polygenic susceptibility to TB and individual variability in drug responsiveness. SNPs in various genes have been linked to the development of TB. This article reviews recent advances in the study of TB-related cytokine gene polymorphisms, including interleukin-6 (IL-6), interferon-γ (IFN-γ), IL-17, IL-35, tumor necrosis factor-α (TNF-α), CC chemokine ligand 1 (CCL1), CCL2, CCL5, macrophage migration mannose-binding lectin (MBL), macrophage inhibitory factor (MIF), and transforming growth factor β1 (TGF-β1) (Table 1), aiming to provide new insights for the clinical diagnosis, treatment, and prognostic evaluation of TB.

**TABLE 1:** A summary of gene polymorphisms related to pulmonary tuberculosis

Gene	Chromosome	Variant	Results	Affected people	Study	References
Interferon-γ (IFN-γ)	12q24	+874A>T (rs2430561)	Associated with reduced risk of PTB	Caucasian	Meta-analysis	(19)
			Not associated with the risk of PTB	Asian		
		+874 T/A (rs2430561)	Associated with increased susceptibility to PTB	African and American	Meta-analysis	(20)
			Associated with more susceptible to PTB and EPTB	East Asian		
		+874 T/A (rs2430561)	A determinant in the resistance or susceptibility to the development of active TB	Tunisian	Case-control	(21)
		AA genotype with IFN-γ+874 T/A	Associated with susceptibility of PTB	Chinese	Case-control	(22)
		CC + CT genotype (rs1861494)	Associated with decreased risk of LTBI	Chinese	Case-control	(23)
Interleukin (IL)						
IL-6	7p21	GA(rs2069837）	Associated with decreased risk of PTB	Chinese	Case-control	(24)
		GG genotype (rs1800796)	Associated with reduced risk to PTB	Chinese	Case-control	(25)
		rs1800795	Associated with susceptibility of PTB	Asian	Meta-analysis	(26)
		rs1800795	Be positive in China (recessive comparison)		Meta-analysis	(27)
			Iran, Pakistan, India(dominant, recessive, and allele comparisons)			
IL-10	1q31-32	−592 A＞C	Associated with decreased risk of PTB	Korean	Case-control	(28)
		−1082 A > G (rs1800896)	Not associated with the risk of PTB	Asian and African	Meta-analysis	(29)
			Associated with increased risk of PTB	Caucasian		
		−1082G/A	Associated with APTB and HHC	Indian	Case-control	(30)
IL-17	6p12	AA genotype and carriers of A allele	Associated with decreased risk of PTB	Brazilian	Case-control	(31)
		IL-17A-152G allele and-152GG genotype	Associate with increased risk of PTB	Northern Spain	Case-control	(32)
		rs763780 allele C and IL17A rs3748067 allele C	Associated with susceptibility of PTB	Asian	Meta-analysis	(33)
		rs22275913	Not associated with the risk of PTB	—		
IL-35	3q25.33 and 19q13.3	rs4740	Associated with the risk of PTB	Chinese	Case-control	(34)
		rs428253, rs4740, and rs9807813	A protective factor for PTB susceptibility	Chinese	Case-control	(17)
Tumor necrosis factor-α (TNF-α)	6p21.3	−308A (G/A + G/G)	Associated with susceptibility of PTB	Iranian	Case-control	(35)
		TNF-308G/A (rs1800629)	Associated with the risk of PTB	Mozambique	Case-control	(36)
		−308 A allele	Associated with increased risk of PTB	Chinese	Case-control	(37)
		TNFA-308G/A	Associated with the risk of EPTB	Brazilian Amazon	Case-control	(38)
		−238G > A	Associated with the risk of PTB	Asian	Meta-analysis	(39)
		−308G > A	Associated with the risk of PTB	African		
		−857 C/T	Associated with decreased risk of PTB	Chinese	Case-control	(40)
		−863 A/C	Associated with susceptibility of PTB			
		−857 T/C	Associated with susceptibility of PTB	Asian	Meta-analysis	(41)
Chemokines						
CCL1	17q11.2-q12	rs2072069	Associated with TBM	Vietnamese population	Case-control	(42)
		rs2072069	Not associated with the risk of PTB	Chinese	Case-control	(43)
CCL2	17q11.2	−2,581 A/G	Not support an association of −2581 A/G with PTB susceptibility	Southeast Iranian	Case-control	(44)
CCL5	17q11.2-q12	rs2734648	Associated with TB recurrence	Chinese	Case-control	(45)
		In1.1T/C (rs2280789)	Associated with early-onset of PTB	Caucasian, of European descent, and (predominantly) ethnically Moldavian	Case-control	(46)
		−403 G > A	Appeared to be linked to susceptibility		Meta-analysis	(47)
Other important cytokines involved in PTB						
Macrophage migration inhibitory factor (MIF)	22q11.2	−173 G/C (rs755622)	Associated with susceptibility of PTB	Asian	Meta-analysis	(48)
		MIF −173C allele	Associated with susceptibility of PTB	Colombian	Case-control	(49)
Mannose-binding lectin (MBL)	10q11.2-q21	MBL (HH) genotype and the H allele	Associated with susceptibility of PTB	Lur population	Case-control	(50)
		54 A/B (rs1800450)	A risk factor for PTB	—	Meta-analysis and systematic review	(51)
		52 A/D (rs5030737)				
		57 A/C (rs1800451)	A protective factor for PTB			
		+4 P/Q (rs7095891)				
Transforming growth factor β1 (TGF-β1)	9q13	C-509T and T869C	Not associated with susceptibility of PTB	Hong Kong Chinese population	Case-control	(52)

### Interferon-γ

The interferon family comprises a group of cytokines that exhibit antiviral activity. Interferons are categorized into three types according to their signaling pathways: type I (including IFN-α, -β, -ε, -κ, and -ω), type II (IFN-γ), and type III (IFN-λ1, -λ2, and -λ3, which are also known as IL-29, IL-28A, and IL-28B, respectively). The IFN-γ gene, approximately 5.4 kb in length, is located on human chromosome 12q24 and comprises four exons and three introns. IFN-γ is mainly produced by activated CD4^+^ and CD8^+^ T cells, as well as natural killer (NK) cells. It acts as an important Th1-like cytokine, activating and regulating CD8^+^ cytotoxic T lymphocytes and mast cells. This makes it vital for antiviral responses and the regulation of cellular immunity (53). The IFN-γ gene contains six polymorphic sites located within the promoter (183G/T, 155A/G) (54), intron 1 (874T/A) (55), intron 3 (2109A/G, 3810G/A), and 3′-end noncoding region (5134G/A)(56). Polymorphisms in the IFN-γ gene may influence disease development by affecting IFN-γ expression and secretion, thereby altering the body’s regulatory antiviral and immune responses.

In recent years, several studies have highlighted a correlation between the IFN-γ +874 polymorphism and the development of pulmonary tuberculosis (PTB); however, these findings are inconsistent. A recent meta-analysis involving 21 case-control studies, which included 281 confirmed PTB patients and 5,186 healthy controls, revealed that the IFN-γ + 874 A > T gene polymorphism is significantly linked to a reduced risk of developing PTB, indicating a protective effect in Caucasian populations. Conversely, this polymorphism was not associated with the risk of PTB in Asian populations (19).

This result suggests that the role of the same genetic polymorphism in PTB susceptibility may vary among different races. Another study confirmed that the IFN-γ +874T/A polymorphism was associated with increased susceptibility to pulmonary infections in African and American populations, whereas East Asian groups were more vulnerable to both pulmonary and extrapulmonary tuberculosis (EPTB). This increased susceptibility is attributed to the frequent presence of AA genotypes (+874 IFN-γ T to A SNP). Conversely, Asians possessing the “A” allele or the AA genotype (+874 IFN-γ T to A SNP) appear to be more susceptible solely to EPTB. The TT genotype was associated with a reduced risk of EPTB in East Asians, although no significant role of the TT genotype was observed in other ethnic groups with EPTB (20). In addition, a study conducted with Tunisian patients revealed that the +874AA genotype was significantly associated with a greater risk of developing active tuberculosis. Conversely, the AT genotype was significantly associated with resistance to the development of both PTB and EPTB (21). Wu et al. demonstrated that the AA genotype of IFN-γ(+874T/A) is considered a susceptibility genotype for TB. The underlying mechanism may be related to a Th1/Th2 imbalance, with patients harboring the AA genotype being more prone to this imbalance. This imbalance can lead to a reduction in the body’s immune function, increasing susceptibility to infection by TB-inducing mycobacteria (22). The +874 T>A SNP in the IFN-γ gene may result in alterations to the NF-κB binding site, leading to variations in the binding affinity of NF-κB transcription factors among individuals with different genotypes. These variations subsequently influence the expression levels of IFN-γ (57). Specifically, the AA genotype is associated with lower IFN-γ expression, likely due to reduced NF-κB binding affinity, whereas the TT genotype is linked to higher IFN-γ expression due to enhanced binding affinity (58). In summary, the IFN-γ +874 T>A polymorphism modulates the binding capacity of NF-κB, resulting in genotype-dependent differences in IFN-γ expression levels. These differences directly impact the strength and intensity of immune responses, highlighting the functional significance of this genetic variation. Collectively, these findings suggest that the presence of the AA genotype may play a role in the development of disseminated forms of TB, with implications for clinical diagnosis and treatment. Regarding other IFN-γ gene polymorphisms, a study in China indicated that the IFN-γ rs1861494 genotype is a risk factor for latent pulmonary tuberculosis. In addition, IFN-γ and IFN-γ receptor 1 polymorphisms did not affect the results of the IFN-γ release assay (23). Therefore, future studies with larger sample sizes from various geographic locations and ethnic groups are needed to assess the relationship between IFN-γ gene polymorphisms and PTB.

### Interleukin

#### IL-6

IL-6, a member of the cytokine family, is composed of two glycoprotein chains: a chain with a relative molecular mass of 80,000 and a chain with a relative molecular mass of 130,000. IL-6 is secreted by various cells in the human body, including Th2 cells and fibroblasts (59). The gene encoding human IL-6 contains four introns and five exons. The human IL-6 precursor is composed of 212 amino acids; after the removal of the N-terminus, which comprises 28 amino acids, a mature IL-6 of 184 amino acids is produced (60). The IL-6 gene is located on chromosome 7p21 and has two types of polymorphisms: high yield and low yield (61). The high-yield type is found in −174G/G and −174G/C gene carriers, resulting in elevated levels of IL-6 in the peripheral circulation; conversely, the low-yield type is associated with −174C/C gene carriers, leading to reduced IL-6 levels in the peripheral circulation (61). SNPs at IL-6 –174, −572, and −634 are found in the peripheral circulation and have been implicated in various diseases (62).

Wu et al. identified the rs2069837 polymorphism of IL-6 in a Chinese Han population. The frequency of the AA genotype was 144 (68.9%) in the tuberculosis group and 129 (64.5%) in the latent tuberculosis group. However, the frequency of GA genotypes in the TB group was significantly lower at 56 (26.8%) than in the latent tuberculosis group at 68 (34.0%). This difference is statistically significant. Compared with AA genotypes, GA genotypes serve as a protective factor in reducing the risk of latent tuberculosis infection (LTBI) developing into PTB (24). This highlights the protective role of the GA genotype in TB progression. Zhang et al. conducted a study with two independent cohorts: an experimental group comprising 495 cases and 358 controls and a validation group consisting of 1,383 cases and 1,149 controls. The findings revealed that the frequency of the G allele in the TB group was significantly lower than that in the control group. In addition, CD14^+^ monocytes isolated from individuals with the protective rs1800796GG genotype produced lower levels of IL-6 than those with the CC and CG genotypes. However, regardless of the rs1800796 genotype, no differences were observed in the production of IL-10 and IL-12p70. They determined that the rs1800796 GG genotype in the IL-6 promoter offers protection against TB by downregulating IL-6 production (25). Consistent with the research findings of Gu et al. (63), the rs1800796 C > G mutation inhibits the transcriptional activity of the IL-6 promoter, leading to a decrease in IL-6 expression. It suggests that the GG genotype may protect against TB by reducing the expression of IL-6. Low levels of IL-6 help reduce the over-activation of the immune system, thereby reducing susceptibility to TB. Future research should further explore the specific mechanism of this gene mutation and its role in TB immune response. Positive results were observed for IL-6 rs1800795 in the PTB and Asian subgroups in a meta-analysis (26). Another meta-analysis showed the rs1800795 polymorphism demonstrated positive associations in China and Iran, Pakistan, and India (27). These findings suggest that the IL-6 rs1800795 polymorphism can serve as a potential genetic marker for assessing the risk of PTB, which is of great significance for understanding the pathogenesis of the disease and identifying high-risk populations.

#### IL-10

IL-10 is secreted predominantly by monocyte macrophages and helper T cells (64). In addition, it is produced by other inflammatory cells, vascular cells, and adipocytes (65). IL-10 has a relative molecular mass of 37,000, while its active form is a noncovalently bonded oligodimer comprising two monomers, each with a relative molecular mass of 18,500 (66). The IL-10 gene is located on chromosome 1 at the 1q31-32 region and spans approximately 4.7 kb, comprising five exons and four introns (67). The human cell surface IL-10 receptor selectively binds to human IL-10 and primarily inhibits monocytes/macrophages, NK cells, T cells, B cells, and mast cells (68). IL-10 exhibits potent anti-inflammatory and immunosuppressive activities. On the one hand, it can inhibit the synthesis and release of inflammatory factors; on the other hand, it can suppress the activation, migration, and adhesion of inflammatory cells, demonstrating synergistic effects with other anti-inflammatory mediators (69).

In the Korean population, the frequency of the “C”-bearing genotype was greater among normal controls (55.8%) than among patients with clinical TB infection (47.1%). One common promoter SNP (IL10-592 A > C) and a prevalent haplotype (ht2[A-C-C-T]) were significantly associated with a reduced risk of TB. Furthermore, it is highly probable that the genetic function of IL10-ht2 is influenced by the IL10-592 A > C variant (28). This agrees with the findings of López-Maderuelo et al. (58). The meta-analysis included 22 eligible studies, including 4,956 cases of PTB and 6,428 healthy controls. The subgroup analysis revealed that the IL-10–1082 A > G polymorphism had no role in PTB susceptibility in Asian and African populations. Interestingly, the dominant model (GG + AG vs. AA) demonstrated an increased risk of PTB in the Caucasian population (29). This may indicate that the IL-10–1082A > G polymorphism has different regulatory effects on immune response and TB susceptibility in different ethnic groups. Joshi et al. selected 150 patients with active pulmonary tuberculosis (APTB), 190 household contacts (HHC), and 150 healthy controls (HC). The results revealed that the GA genotype was associated with a 2.3-fold greater risk in APTB patients and a 3.7-fold higher risk in HHC compared to HCs. Both the G and A alleles were significant in APTB patients and HHC, whereas the A allele was positively associated with the risk of disease in both APTB patients and HHC. The G allele was negatively associated with both APTB patients and HHC. And also revealed a strong correlation between the IL-6 genotype and the IL-10 genotype. Furthermore, the interaction between these genes indicates a high risk (30). In future studies, gene interactions may be utilized to identify individuals at high risk for TB.

#### IL-17

IL-17A is a member of the IL-17 family and functions as the signature cytokine of helper T cells 17 (70). It can be secreted by various immune cells, including NK cells, CD8^+^ T cells, γδ T cells, and specific epithelial and vascular endothelial cells (71). The IL-17 protein (molecular weight of 15 kDa) consists of 150 amino acids, and its gene is located on human chromosome 6p12 (72). The literature confirms that NF-κB activator 1 and TNF receptor-associated protein 6 are key linkers for IL-17F, with Act1 primarily acting on IL-17 through the IL-17RA receptor. In addition, TRAF6 is directly involved in the IL-17 signaling pathway because it functions as an E3 ubiquitin ligase (73). IL-17 exerts its biological effects primarily through two pathways: the mitogen-activated protein kinase pathway and the NF-κB-DNA pathway (74). In the southern Brazilian population, the −197A allele in the IL-17A 197A > G polymorphism was significantly more prevalent in the control group than in PTB patients. In addition, logistic regression analysis revealed a reduced risk of PTB development among individuals with the AA genotype and those carrying the A allele (AG + AA). Furthermore, the −197A allele associated with TB corresponds to elevated levels of IL-17A release (31). Research has indicated that elevated IL-17A production may be advantageous in preventing mycobacterial infections by promoting the formation of mature granulomas and inhibiting disease progression (75, 76). However, in northern Spain, patients with PTB in this study presented elevated levels of IL-17, with the IL-17A-152G allele and −152GG genotype linked to an increased risk of PTB. This association may be influenced by other adjacent functional IL-17A SNPs affecting IL-17 expression (32). A meta-analysis revealed that exposure to the IL-17F rs763780 allele C and the IL17A rs3748067 allele C may be linked to susceptibility to TB in Asian populations. However, no significant association was observed between the IL-17A rs22275913 polymorphism and TB susceptibility (33). These results are consistent with those of a study conducted in China on IL-17A rs3748067 and PTB (77). The IL-17A rs3748067 polymorphism may serve as a potential genetic marker for PTB.

#### IL-35

IL-35, a member of the IL-12 family, is secreted by regulatory T cells and other cells in response to inflammatory events in the intestinal tract. IL-35 inhibits the activation of monocyte-macrophage functions and regulates the expression of various intrinsic immune cytokines, thereby exerting an immunosuppressive effect (78). Unlike IL-12 and other cytokines, IL-35 promotes the expression of FoxP3 and the secretion of IL-10 by CD4^+^CD25^+^ T cells, thereby mediating the suppression of effector T cells, including Th1 and Th17 cells (79). Furthermore, IL-35 transforms proinflammatory CD4^+^ T cells into regulatory T cells (iTregs35), which express IL-35 but not IL-10, TGF-β or FoxP3. These cells can effectively inhibit the onset and progression of several autoimmune diseases (80).

IL-35 is characterized by a newly discovered dimeric structure composed of two subunits, EBI3 and p35, and is predominantly expressed in Treg cells (81, 82). The gene IL-12A, which encodes p35, is located on human chromosome 3q25.33 and comprises seven exons, whereas the coding gene for EBI3, which is located on human chromosome 19q13.3, consists of five exons (83). Zheng et al. conducted a study involving 435 patients with PTB and reported that the EBI3 rs4740 gene polymorphism is associated with susceptibility to PTB, with EBI3 expression levels correlated with disease severity (34). The SNP rs4740 mutation may affect the structure or function of the EBI3 gene (84). In addition, this mutation interferes with T cell recognition (85). Therefore, the rs4740 polymorphism in EBI3 may be a key genetic factor regulating T-cell-mediated immunity, affecting the susceptibility of TB and the severity of the disease. Gao et al. demonstrated that the GAC haplotype for rs4740, rs428253, and rs9807813 in the IL-35 gene acts as a protective factor against PTB susceptibility in a Chinese Han population. In addition, the IL-35 rs428253 GC genotype, as well as the rs4740 AA genotype and A allele, was significantly related to hypoproteinemia in PTB patients. Furthermore, a lower frequency of the IL-35 rs568408 GA genotype was associated with drug-induced liver injury in patients with PTB (17). Overall, these findings emphasize the importance of IL-35 gene polymorphism in regulating susceptibility to PTB and clinical outcomes in PTB patients, including complications such as hypoalbuminemia and drug-induced liver injury. This highlights the potential of using IL-35 polymorphism as a biomarker for the severity of PTB disease and treatment response.

#### Tumor necrosis factor-α

Tumor necrosis factors (TNFs) are cytokines with various biological activities in the body and are classified into two types based on their origins: TNF-α and TNF-β. These cytokines are secreted by monocytes, macrophages, and lymphocytes, and these molecules can stimulate their synthesis and enhance the production of other cytokines, including IL-1 and IL-6. TNFs increase the expression levels of HLA-I and HLA-II antigens, facilitate the proliferation of both B and T lymphocytes, and increase immunoglobulin synthesis. As a result, they play a crucial role in the induction of inflammation and the modulation of immune responses (86). Mutations in the TNF-α gene, primarily located on chromosome 6p21.3 (87).

TNF gene polymorphisms are characterized by base changes in the gene, including single base substitutions, insertion of cytosine (C) in the first exonic region at position +70, substitution of guanine (G) for adenine (A) in the first intronic region at position +489, and deletion of G in the first intronic region at position +691. Notably, adenine (A) is substituted for guanine (G) at the transcription start site of the promoter region (position −308), resulting in the loss of the recognition site for the restriction endonuclease NcoI due to this mutation. Consequently, this polymorphism manifests in changes in restriction fragment length (88). The host response to *M. tuberculosis* is mediated by cellular immunity (89). TB mainly affects lung macrophages, with Th1-mediated immunity recognized as the protective mechanism against *M. tuberculosis*. In this context, cytokines such as interferon-gamma and tumor necrosis factor play essential roles in the immune response, limiting the dissemination of bacilli by facilitating granuloma formation to contain the mycobacteria (90). These observations indicate that TNF-α is essential for both the development and preservation of granulomas in PTB. The TNFA-308 gene polymorphism is associated with TB in populations from China, Iran, and Mozambique (35–37). Moreover, the frequency of the TNFA-308G/A polymorphism genotype has been associated with EPTB in a population from the Brazilian Amazon (38). A meta-analysis indicated that in Asian populations, TNF-238G > A is significantly associated with PTB, whereas TNF-α−308G > A is associated with PTB in African populations (39). These findings indicate that the TNFA-308 polymorphism could serve as a significant biomarker. Consequently, it is crucial to conduct studies with larger sample sizes to validate the role of the TNFA-308G/A polymorphism in TB susceptibility within the population. Conversely, Ma et al. conducted a hospital-based case-control study involving 543 patients and 544 controls. Multivariate logistic regression analysis suggested that the TT genotype of the 857C/T polymorphism in the TNF gene may serve as a protective factor for PTB, whereas the AA genotype of the 863A/C polymorphism could represent a risk factor for PTB and be associated with clinical severity (40). In addition, a meta-analysis indicates that the TNF-α−857T/C polymorphism is linked to increased susceptibility to PTB among Asian populations (41). While both articles reported the association between the TNF-α−857 polymorphism and Asian populations, it remains unclear whether this association exists in other ethnic groups. Therefore, additional research is necessary to explore this issue further.

#### Chemokine

Chemokines, often referred to as chemotactic cytokines, are a group of small proteins consisting of 70–125 amino acids with molecular weights between 8,000 and 10,000 Daltons that are capable of inducing the migration of various cell types. The disulfide bond that stabilizes the structure of chemokine monomers features a central triple-stranded β-fold, an overlying C-terminal α-helix, and a short unstructured N-terminus, which is essential for receptor activation (91). Over 50 chemokines have been discovered, with the majority featuring four conserved cysteine residues. They are categorized into four subfamilies—CXC, CC, C, and CX3C—according to the arrangement of the first two cysteine residues (where “C” denotes cysteine and “X” represents any amino acid). The CXC and CC subfamilies are the most prominent among these (92). Research has indicated that the expression of chemokines and their receptors is influenced by polymorphisms in their respective genes (93–95).

#### CCL1

CCL1 is a gene located within the CCL subfamily chemokine gene cluster on chromosome 17q11.2-q12 (96). It is secreted by activated T cells and binds to the chemokine CC motif receptor 8 (CCR8), which is known to be involved in immune regulation and inflammatory processes (97). CCL1 is expressed and secreted by monocytes, and its receptor, CCR8, is present on the surface of Th2 cells and Tregs. Through the activation of CCR8, CCL1 induces a Th2-type cellular immune response, which subsequently suppresses Th1-type cellular immunity and reduces the ability of the immune system to eliminate *M. tuberculosis*. Two studies have investigated the rs2072069 polymorphism of CCL1. Thuong et al. analyzed peripheral blood samples from 114 patients with tuberculous meningitis (TBM) and 159 patients with PTB, while the control group comprised umbilical cord blood. They utilized whole-genome testing for the first time to identify patients with various clinical forms of TB and discovered that the rs2072069 polymorphism in the CCL1 gene was associated with TBM in the Vietnamese population (42). By contrast, a study conducted in China using venous blood samples revealed no association between the rs2072069 polymorphism in the CCL1 gene and the various clinical forms of TB (43). The discrepancy between the studies by Thuong et al. and the Chinese study regarding the rs2072069 polymorphism in the CCL1 gene may be due to several factors. These include genetic differences between the populations, with varying allele frequencies and immune responses, differences in sample types (peripheral blood vs. venous blood), and study design (whole-genome testing vs. targeted polymorphism analysis). In addition, the sample size and clinical form of TB (TBM vs. PTB) might contribute to the observed differences. Further research with larger, diverse cohorts is needed to clarify the role of CCL1 polymorphisms in TB susceptibility.

#### CCL2

MCP-1, also referred to as CCL2, is a key member of the C-C chemokine family. The MCP-1 gene is located at 17q11.2–12 and comprises three exons and two introns (98). MCP-1 has a strong chemotactic effect on monocytes and macrophages, exerting its activity by specifically binding to CCR2, which recruits and activates monocytes and macrophages at inflammatory sites (99). Numerous studies have established that the expression level of MCP-1 is linked to the occurrence, progression, and prognosis of various diseases. A case-control study involving 142 patients with PTB and 166 healthy subjects revealed no evidence supporting an association between the −2581A/G polymorphism of CCL2 and PTB susceptibility (44).

#### CCL5

CCL5, a member of the CC chemokine family, is recognized as a major chemokine primarily involved in immunoregulatory and inflammatory activities owing to its ability to recruit, activate, and costimulate T cells and monocytes (100, 101). The human CCL5 gene is located at 17q11.2-q12 and encodes a chemokine protein consisting of 91 amino acid residues, with a molecular weight of 8 kDa. It shares high homology with macrophage inflammatory protein-1α and macrophage inflammatory protein-1β (102). Two single nucleotide polymorphisms, rs2107538 and rs2280788, in the promoter region of the CCL5 gene, regulate its transcriptional activity and are associated with human immunodeficiency virus infection (103). However, few studies have examined the correlation between the CCL5 gene and PTB, and existing reports have highlighted certain population differences. Liu et al. reported that the allelic frequency of rs2734648-G was significantly higher in the tuberculosis patient group, especially among those with PTB, than in the control group. Furthermore, carriers of the rs2734648-GG genotype presented a 2.382-fold increased risk of susceptibility to PTB in a recessive inheritance model. In addition, rs2734648 was significantly associated with TB recurrence (45). SNPs in the CCR5 promoter may have different effects on transcription factor binding (104). Research has shown that the G to T substitution in rs2734648 is associated with differences in the binding ability of the NF-κB transcription factor family (105). The rs2734648-G allele may enhance the transcriptional activity of CCR5 by altering the binding mode of NF-κB transcription factors, leading to excessive migration and activation of immune cells, thereby increasing susceptibility and recurrence risk of TB. Another study demonstrated that the CCL5 In1.1T/C polymorphism was significantly associated with early-onset TB in patients <30 years of age (using a dominant model) and those <40 years of age (also using a dominant model). The case-only analysis revealed that carriers of the C-allele experienced an earlier onset of TB than TT homozygotes, with an average age of onset of 36.14 years versus 40.13 years (46). A meta-analysis assessed the relationship between the CCL5-403 G > A polymorphism and TB risk, indicating that this polymorphism is associated with increased susceptibility to the disease (47). CCL5 plays a crucial role in the immune response against *M. tuberculosis*, particularly in enhancing the immune response by recruiting monocytes to the site of infection (106). Research reveals the presence of the CCL5-403G > A polymorphism is associated with a decrease in serum levels of CCL5 (107), suggesting that low levels of CCL5 may lead to insufficient recruitment of immune cells, thereby reducing the host’s immune clearance ability against *M. tuberculosis*, making the infection more likely to progress to TB.

### Other cytokines related to PTB

#### MIF

MIF is a multifunctional proinflammatory cytokine that promotes the secretion and expression of various inflammatory factors. It is widely expressed in monocyte macrophages, endocrine cells, endothelial cells, epithelial cells, and vascular smooth muscle cells. The human MIF gene is located on chromosome 22q11.2 and consists of three exons and two introns, with the exons measuring 107, 172, and 66 base pairs, while the introns are 188 and 94 base pairs long, respectively. Compared with 114 amino acids, MIF has a molecular weight of approximately 12.5 kDa (108). Due to the high sequence homology of MIF exon structures and DNA sequences across the evolutionary phylogenies of different species, MIF exhibits similar primary structures in mammals, protozoa, and plants (109, 110). As a pleiotropic protein, MIF exists as a monomer, dimer, or trimer and functions in various capacities, including as a cytokine, chemokine, enzyme, hormone, and molecular chaperone. Notably, the number of microsatellite repeats at the 5′ end of the MIF gene regulates the level of MIF expression, which is implicated in the onset and progression of numerous diseases (111).

Tong et al. discovered that individuals with the variant C allele in the MIF gene have increased susceptibility to PTB. Subgroup analyses revealed that the MIF-173G/C gene polymorphism is specifically associated with PTB susceptibility in Asian populations (48). Studies have shown that the G to C mutation at the MIF-173 site may significantly enhance the transcriptional activity of MIF genes by forming a new transcription factor activator Protein-4 binding site, thereby increasing the expression level of MIF (112). According to the research of Tong et al. (48), individuals carrying the C allele show a higher susceptibility to PTB in Asian populations. This susceptibility may stem from increased MIF expression caused by the C allele, leading to excessive pro-inflammatory response or immune imbalance, weakening the host’s ability to control *M. tuberculosis*. Therefore, the MIF-173G/C polymorphism plays an important role in PTB susceptibility by altering gene expression levels. In addition, multivariate analysis of another group of studies showed that the MIF-173C allele is linked to the disease in a dominant manner. By contrast, no alleles in the MIF-794 CATT microsatellite were found to be associated with an elevated risk of TB (49). This may be because the MIF-794 microsatellite polymorphism has a relatively small regulatory effect on MIF expression or function, and further research will help reveal the specific mechanisms of MIF gene polymorphism in TB susceptibility.

#### MBL

MBL is a calcium-dependent sugar-binding protein that is structurally akin to C1q and plays an essential role in the nonspecific immune response by binding to mannose residues (113). It is abundantly found in the liver and blood of humans and serves as a critical component of the innate immune system. Serum MBL levels are influenced primarily by polymorphisms in the first exon and promoter region of its structural gene (MBL2). The MBL pathway is crucial for pathogen recognition by the innate immune system, as it detects peptidoglycan from gram-positive bacteria via its C-type lectin structural domain and modulates the release of cytokines and chemokines (114).

The human MBL gene is located between 10q11.2-q21 and contains one 5′ promoter region, four exons, and three introns (115). Codons 52, 54, and 57 in exon 1 of MBL2 are the most common structural mutations. Three single nucleotide polymorphism (SNP) sites exist in the promoter region of the MBL gene: H/L at −550, X/Y at −221, and P/Q at +4. These loci usually form specific haplotypes and are in linkage disequilibrium with the exon sites. To date, seven haplotypes—LYPA, B, HYPA, HYPD, LXPA, LYQA, and LYQC—have been confirmed (116).

One study revealed that the MBL (HH) genotype and the H allele are linked to susceptibility to TB in a Lur population in Iran, with a significant association observed for the L allele in relation to resistance against PTB. Specifically, compared with the control, the HH genotype and H allele increased PTB risk by 1.909-fold and 1.525-fold, respectively, whereas the L allele was associated with a 0.656-fold reduction in PTB risk (50). In addition, Tong et al. conducted a meta-analysis and reported that the MBL-2 rs1800450 (54 A/B) and rs5030737 (52 A/D) polymorphisms are associated with a heightened risk of PTB. Conversely, the MBL-2 rs1800451 (57A/C) and rs7095891 (+4 P/Q) polymorphisms were identified as protective factors against PTB (51), this reveals the dual role of different polymorphisms in the MBL-2 gene in susceptibility to TB. This reflects the complexity of immune response and the decisive role of genetic variation in TB susceptibility.

#### TGF-β1

TGF-β1 is a highly conserved polypeptide cytokine that plays a crucial role in regulating various biological processes in immune cells, such as differentiation, chemotaxis, proliferation, and activation. During TB, TGF-β1 is produced in significant amounts at the site of active *M. tuberculosis* infection (117). It is secreted by monocytes stimulated by *M. tuberculosis* or arabinopeptide and is present in the TB patients. The overexpression of TGF-β1 in tuberculosis foci may be linked to transforming growth factor production by monocyte macrophages induced by *M. tuberculosis* and its active components (118). TGF-β1 can promote the intracellular growth of *M. tuberculosis*, whereas antibodies or natural inhibitors can reduce this growth (119).

The TGF-β1 gene is located on chromosome 19q13 and comprises seven exons and six introns (120). The concentration of TGF-β1 in human blood is regulated by genes, and polymorphisms in this gene correlate with the function and levels of the TGF-β1 protein (121). In a study involving a Hong Kong Chinese population, the TGF-β1 gene polymorphisms C-509T and T869C were not significantly associated with susceptibility to TB. However, elevated plasma levels of TGF-β1 may also contribute to the development and progression of this disease (52). Indicating that while genetic variations in TGF-β1 may not play a critical role in susceptibility, the protein itself might influence the disease’s pathophysiology. Therefore, while genetic variations in TGF-β1 might not be a major risk factor for TB susceptibility in this population, the role of TGF-β1 as a mediator in the disease’s progression should not be overlooked. Further research could explore the mechanisms by which elevated TGF-β1 levels contribute to the progression of TB and whether they might represent a therapeutic target for managing the disease.

### Conclusions

TB is a multifactorial chronic infectious disease with an increasing incidence worldwide in 2021 (9). Research into the pathogenesis and treatment of TB is crucial. Research shows that an individual’s drug treatment response is closely related to genetic factors (122). In pharmacogenomics, genes encoding drug-metabolizing enzymes, drug transporters, and drug targets are associated with drug concentration and drug action, and their polymorphism may affect drug response, thereby affecting the efficacy and safety of drug treatment (123, 124). While the underlying mechanisms of TB are still not fully understood, an increasing number of studies suggest a complex genetic susceptibility to the disease. Therefore, identifying genetic markers and investigating the roles of related genes in TB pathogenesis are essential for effective prevention and treatment strategies. This paper examines the polymorphisms of cytokine genes linked to PTB and summarizes the roles of cytokines—including interferons, chemokines, tumor necrosis factor, and interleukins—in the diagnosis of TB patients. These insights will provide a theoretical basis for understanding disease mechanisms, treatment approaches, and drug development. However, before using genome data as a treatment tool for tuberculosis patients, several key issues and technologies must be solved, including functional verification of gene-phenotype association, development of personalized treatment schemes, ethical and privacy issues, data integration and interpretation, etc. Future research should focus on expanding sample sizes and exploring racial differences to deepen our understanding of gene applications, as well as studying gene-environment interactions and the potential of relevant genes as therapeutic targets. In conclusion, the association between cytokine polymorphisms and TB susceptibility highlights the potential for these genetic variations to serve as biomarkers for identifying high-risk populations. While further research is required, these findings may eventually inform strategies for TB prevention, diagnosis, and personalized treatment.
